# A Path Toward Systemic Equity in Life Cycle Assessment and Decision-Making: Standardizing Sociodemographic Data Practices

**DOI:** 10.1089/ees.2021.0375

**Published:** 2022-09-15

**Authors:** Joe F. Bozeman, Erin Nobler, Destenie Nock

**Affiliations:** ^1^ Civil and Environmental Engineering, Public Policy, Georgia Institute of Technology, Atlanta, Georgia, USA.; ^2^ Geography, Planning and Design, University of Colorado Denver, Denver, Colorado, USA.; ^3^ Civil and Environmental Engineering, Engineering and Public Policy, Carnegie Mellon University, Pittsburgh, Pennsylvania, USA.

**Keywords:** critique, ethnicity, race, review, social, sociodemographic, socioeconomic status

## Abstract

Social equity has been a concept of interest for many years, gaining increased focus from energy and environmental communities. The equitable development, collection, and reporting of sociodemographic data (e.g., data related to socioeconomic status, race, and ethnicity) are needed to help meet several of the United Nations Sustainable Development Goals (i.e., Affordable and Clean Energy; Reduce Inequalities; Peace, Justice and Strong Institutions; and Partnerships for the Goals). Yet, there has not been a consolidation of relevant concepts and application framing in energy and environmental life cycle assessment and decision-making practices. Our study aims to help fill this gap by consolidating existing knowledge on relevant equity applications, providing examples of sociodemographic data needs, and presenting a path toward a more holistic equity administration. In this critique, we present a framework for integrating equity in energy and environmental research and practitioner settings, which we call systemic equity. Systemic equity requires the simultaneous and effective administration of resources (i.e., distributive equity), policies (i.e., procedural equity), and addressing the cultural needs of the systematically marginalized (i.e., recognitional equity). To help provide common language and shared understanding for when equity is ineffectively administered, we present ostensible equity (i.e., when resource and policy needs are met, but cultural needs are inadequately met), aspirational equity (i.e., when policy and cultural needs are met, but resources are inadequate), and exploitational equity (i.e., when resource and cultural needs are met, but policies are inadequate). We close by establishing an adaptive 10-step process for developing standard sociodemographic data practices. The systemic equity framework and 10-step process are translatable to other practitioner and research communities. Nonetheless, energy and environmental scientists, in collaboration with transdisciplinary stakeholders, should administer this framework and process urgently.

## Introduction

There has been a large call to action for including equity, equality, and social justice into technical engineering decisions. This call involves addressing energy transitions, environmental justice, and racial inequity challenges (Biden, 2021).

For clarity, energy transitions refer to jurisdictions (e.g., nations, regions, and locales) that are attempting to transform or develop their energy sector away from fossil fuels to one driven by zero-carbon energy sources. These transitions have been influenced by factors such as price spikes in fuel resources, depletion of energy resource availability, and environmental harm from energy sources that have historically ignited social unrest (Solomon and Krishna, 2011; Diotalevi and Burhoe, 2017). It is important to note that administering just energy transitions align with the intent of the United Nations Sustainable Development Goal (SDG) of Affordable and Clean Energy.

In a National Renewable Energy Laboratory (NREL) workshop, held in April of 2021 over 3 days, transdisciplinary scientists (i.e., social scientists, engineers, community leaders, and urban planners) came together to build understanding on the social, institutional, and cultural dimensions of energy transitions (Romero-Lankao *et al.*, 2021). Key themes that emerged from the workshop included ways forward to address challenges in scale and spatiality, systems thinking and decision-making, solution framing, just energy transitions, and structural barriers to participation.

This article, compelled by the proceedings of this NREL workshop, argues for the standardization of sociodemographic practices from an energy and environmental life cycle assessment (LCA) and decision-making perspective, as a critique. It further recognizes that systemic equity can only be realized when data practices properly match the diverse dispositions of society.

### Life cycle decision support

Effective decision-making in energy and environmental systems is contingent upon the incorporation of robust analytical tools and comprehensive data collection approaches (Finnveden, 2000; Wang *et al.*, 2009; Earles and Halog, 2011). One of the more prominent analytical tools used in decision-making for energy and environmental systems is LCA (Lazarevic and Martin, 2016; Kaab *et al.*, 2019; Mistretta *et al.*, 2019).

While LCA is particularly important in decision-making since it is a tool that facilitates system and product comparison over the entire life cycle (e.g., cradle-to-grave assessments) (Finnveden, 2000; Klöpffer, 2012), it often misses the human and social dimension (Hellweg and Milà i Canals, 2014). LCA studies tend to focus on the technical aspects of technology impact. For example, even when focused on distinct life cycle stages, energy systems analysis may use air emissions as a metric without considering the communities affected by associated technologies (Jordaan *et al.*, 2021).

Although consistently focused on technical content, LCA practically involves social and subjective components whether explicit or not. For instance, the subjectiveness (e.g., modeler's bias) involved in attributional LCA implementation may be covert. Attributional LCAs incorporate functional units for system inputs and outputs, often in the form of some average or estimated value, according to a normative rule (Earles and Halog, 2011). A normative rule is an ethical position that is linked to system delimitation, in that outcomes are perceived as a “good” association or “bad” dissociation (Ekvall, 2000; Weidema *et al.*, 2018).

Social LCAs (S-LCAs), on the other hand, are explicit in their incorporation of social and subjective components (Zamagni *et al.*, 2011). Although clearly defining S-LCA is difficult given that it is an evolving concept and a relatively new LCA methodology, it quantifies the social impacts of system inputs and outputs to provide decision support (Benoit Norris, 2013).

Regardless of what type of LCA style is used, life cycle activities and their associated functional unit(s) ultimately have decision-making implications. Any decision maker that uses LCA findings that exclude the implications of their own actions, while including those from other actors, is socially irresponsible (Weidema *et al.*, 2018). This suggests that all LCAs involving sociotechnical components that will ultimately affect communities (e.g., air pollution) should strive to incorporate the decision-making implications for community stakeholders as a normative practice. We build on this idea, with a focus on addressing the data challenges for groups that have historically been omitted, systematically marginalized, or generalized in a manner that has kept them from influencing LCA and decision-making activities.

### Data practices

Data collection, aggregation, and processing are particularly important in decision-making since forecasting future behavior and technological impacts rely on the incorporation of existing quantitative or qualitative data. Data collection approaches can range from coalescing publicly available data to attaining proprietary data from private companies using nondisclosure agreements. The act of aggregating, normalizing, and qualifying these data is often time-consuming and inconsistent. Validating the efficacy of these collected data may be inhibited by the lack of useful historical data, particularly where equity is concerned due to large oversight of the social dimension in LCA.

Using sociodemographic data is vital in holistic and equitable energy and environmental decision-making. Recent studies suggest that applicable data collection activities are not truly comprehensive unless they allow for distinction along the lines of race, ethnicity, socioeconomic status (SES), and spatiality (Fortier *et al.*, 2019; Subramanian *et al.*, 2021; Tong *et al.*, 2021). Yet, collecting and reporting on this sort of data are challenging given the intersection of social, economic, and technological factors (Baker *et al.*, 2021).

The challenges involved in sociodemographic data practices are wide ranging. They include addressing the uncertainty in self-reported data (Bozeman *et al.*, 2020; Baltruszewicz *et al.*, 2021); geographic and spatial limitations (Nock and Baker, 2019; Pfeiffer *et al.*, 2021); lack of robust racial, ethnic, and socioeconomic data access (Reames, 2016; Bozeman *et al.*, 2019); individual privacy concerns when data are at a high resolution (Lisovich and Wicker, 2008; McLean, 2016); and structural barriers that effectively disincentivize the publishing and reporting of racial and ethnic differences (Winnifred, 2010; Barber *et al.*, 2020; Stevens *et al.*, 2021).

Furthermore, similar challenges may arise in cases where completed LCAs, from previous studies, are used in subsequent decision-making analyses. For example, Klein and Whalley (2015) performed an LCA on energy technologies in the United States that was later used by Nock and Baker (2019) for a regional analysis in the northeast region of the United States.

Because the data used in these studies were performed at large spatial scales, they provided regional insights but did not show how technology deployment and its associated externalities impact vulnerable communities.

Sociodemographic data practices may be impractical to execute in some cases. Circumstances where the LCA spatial scale does not lend itself to sociodemographic data administration, as was the case in Nock and Baker (2019), may arise. In other cases, it is the overall LCA framing that may undermine administration (e.g., an in-development laboratory technology). This may stem from inapplicability or the unavailability of sociotechnical data for similar technologies or spatial scales. Nevertheless, the conditions of sociodemographic data administration should be justified or explicitly noted in such cases.

Omitting or underrepresenting sociodemographic data has contributed to inequality through ineffective decision-making activities. Extreme inequality is a core impediment to reaching energy and environmental sustainability goals (e.g., the Reduce Inequalities SDG) (Doyle and Stiglitz, 2014; Freistein and Mahlert, 2016; Niessen *et al.*, 2018). Inequality is considered “extreme” once associated factors evidently limit economic growth and contribute to the destabilization of social and political systems (Doyle and Stiglitz, 2014). Developing methods and policies that address extreme energy and environmental inequality is reliant upon access to sociodemographic data across temporal and spatial scales.

### Study aims

As a step toward standardizing sociodemographic data practices for LCA and decision-making in energy and environmental research and practitioner settings, we critique equity concept**s**, present a framework for integrating equity, provide example sociodemographic data needs, and provide guidance for developing corresponding standards and conditions. These efforts align with our aim to consolidate existing knowledge on relevant equity concepts and to present a path toward systemic equity within these subfields. The remainder of this article has three main sections. We begin with the Needs Qualification section, followed by the Standardization of Data Practices section, and end with the Conclusion and Discussion section.

## Needs Qualification

### Energy and environmental conjoining justification

We begin with a justification for conjoining the energy and environmental subfields. There are certainly several distinctions between energy and environmental research and practitioner norms and activities. For instance, a prominent certification for energy practitioners is the Certified Energy Manager credential, whereas environmental practitioners who manage hazardous waste seek annual Resource, Conservation, and Recovery Act and triennial Department of Transportation credentials. From a researcher perspective, energy studies may focus their outcomes on the energy use index. On the contrary, an environmental study may focus its outcomes on air and land implications that derive from energy use activities. In either case, whether it is from a practitioner or research perspective, distinct skill sets are required to attain meaningful results.

Yet, the noted distinctions in practitioner and research activities do not necessarily translate into core sociodemographic data practices. In many cases, energy and environmental researchers use the same or similar source data to perform LCA or decision-making studies. There are established subfields that formally join energy and environmental research practices such as those that investigate the food-energy-water and energy-water nexuses. This merging of disciplinary practices is an apt response by the scientific community to develop transdisciplinary hubs aimed at addressing the complex and wicked challenges of our time (e.g., meeting the SDGs), addressing climate change mitigation, adaptation, and migration issues (Bai *et al.*, 2018).

### Delineating equity

Social equity has been a concept of interest for many years, gaining notoriety in the 1960s (Pulido, 1996; Guy and McCandless, 2012). Despite this growing popularity, social equity has not been thoroughly investigated at the intersection of technology assessment and environmental implications for long. For instance, the majority of studies exploring environmental stewardship and social equity have been published starting only in the year 2009 (Friedman *et al.*, 2018). It follows that equity, its key associated concepts (e.g., justice), and their applications continue to evolve.

The literature on energy, environmental justice, and equity shows that associated concepts and definitions have evolved over time without consensus on clear delineations (Ikeme, 2003). While these terms have gained increasing attention (Baker *et al.*, 2021), there is still no globally agreed upon definition for energy justice, environmental justice, and equity (Jenkins *et al.*, 2020). This lack of consensus can create competing and conflicting interpretations. For example, the US Environmental Protection Agency defines environmental justice primarily in terms of procedural justice, by focusing on the fair treatment and involvement of all people in the creation, execution, and enforcement of environmental laws, policies, and regulations (EPA, 2021). Yet, environmental laws can differ between countries or regions (Balsiger, 2011), which could, in turn, create differences in the enforcement of such measures.

Scholars and advocates have expanded equity and justice definitions and concept delineations to include energy, climate factors, and the perspectives of historically excluded voices (Jenkins *et al.*, 2016; Heffron and McCauley, 2018; Baker *et al.*, 2019; Carley and Konisky, 2020). One article presents an energy justice framework that evaluates equity implications at the intersection of the location of injustices, who has been ignored, and the process for rectifying historical injustices (Jenkins *et al.*, 2016). In another study, their definitions were narrower and focused on distributional equity, calling for the incorporation of distributional considerations in energy and climate policy development such that everyone can benefit equally from job training and economic development (Carley and Konisky, 2020). Others have highlighted that energy and environmental justice definitions overlap, but generally refer to achieving social, economic, and health equity (Heffron and McCauley, 2018; Baker *et al.*, 2019).

Given the continued evolution of energy and environmental justice concepts, we sought to use equity and justice delineations that were clear, broad enough for wide interpretation, and contextualized by energy and environmental influences. Romero-Lankao and Nobler (2021) developed a unifying framework for energy, climate, and environmental justice and equity terminology centered on five tenets (i.e., distributional, procedural, recognition, cosmopolitan, and restorative justice). Moreover, they explained that equity and justice are different concepts: that is, *equity* refers to being fair and unbiased as a function of an organization or system, whereas *justice* primarily involves removing barriers that prevent the implementation of equity.

In the present study, we use the equity definition from Romero-Lankao and Nobler (2021) since it provides clear delineation between equity and justice concepts, is broad enough for wide interpretation, and is contextualized by energy and environmental influences.

### Systemic equity framework

To help establish a framework for integrating systemic equity in LCA and decision-making, we provide a useful synthesis of relevant justice and equity concepts. The core conceptual tenets of energy justice are distribution, recognition, and procedure (Jenkins *et al.*, 2016). Nevertheless, these concepts deserve further development given there is continued deliberation over universal applicability and framing (Sovacool *et al.*, 2016; Lau *et al.*, 2021). For instance, Sovacool *et al.* (2019) presented energy injustices using spatial scale designations (i.e., micro, meso, and macro). Yet, in Jenkins *et al.* (2018), energy justice is framed as a multilevel perspective on sociotechnical systems (i.e., niche, regime, and landscape).

These are examples of spatial framing that have elements which overlap, conflict, or need refining to reach some level of universality. Instead of revisiting spatial framing in this regard, we synthesize distributive, procedural, and recognitional equity by providing example applicability for sociodemographic practices in varying types of organizations or systems.

*Distributive equity* refers to the act of providing tangible resources to a person or group in an unbiased and fair manner. An example for how distributive equity interplays with sociodemographic data practices is the fair distribution of grant funds between black, Hispanic, and nonminority researchers. There are racial funding disparities in National Institutes of Health proceedings, especially when comparing qualified black and white principal investigators (Stevens *et al.*, 2021). Black principal investigators award probability was ∼55% of their white counterparts in the periods of 2000–2006 and 2014–2016. Inequitable distribution of research funds systematically inhibits the development and reporting of sociodemographic data in various ways.

Specifically, funding is needed to facilitate the collection of racial and ethnic data where they currently do not exist or are undercollected. Members of systematically marginalized subgroups can provide insights into where, what, and how to collect meaningful data for their respective subgroups, which might otherwise be missed by nonminorities or those less familiar with subgroup idiosyncrasies.

*Procedural equity* refers to the act of using procedures and decision-making activities that facilitate the allocation of resources in an unbiased and fair manner. Its successful application relies on systematically addressing inequitable power structures, the biases of system stakeholders, and information sharing challenges in decision-making (Hartman *et al.*, 1999; Terpstra and Honoree, 2003; Adeyeye *et al.*, 2019). Some might argue that objectivity in this context means collecting data or presenting outcomes with a one-size-fits-all approach to not single out any one racial or ethnic subgroup. For instance, past energy and environmental system studies have presented outcomes that center on generic population results or just SES, while omitting or generalizing race and ethnicity implications (Sobal, 1991; Havard *et al.*, 2009).

However, generalizing SES as a proxy for race and ethnicity can be misleading (Havard *et al.*, 2009). This can take form in activities such as recoding disparate racial and ethnic data to represent as “white *versus* other” as well as failing to develop or report on racial and ethnic outcomes when possible (Hearst *et al.*, 2013; Conrad *et al.*, 2018). Studies such as these may yield important discoveries but should be viewed as addressing only part of the core sociodemographic landscape. In contrast, other studies have shown that race, ethnicity, and SES are distinct social frameworks with unique system implications, although these sociodemographic groups can overlap in important ways (Williams, 1996; Wood and Horner, 2016; Hamann *et al.*, 2018; Gurram *et al.*, 2019).

*Recognitional equity* refers to addressing the psychological, emotional, and cultural needs of the systematically marginalized where bias and disadvantage are embedded or evident. This equity concept has received little attention in environmental stewardship research compared to distributive and procedural equity matters (Friedman *et al.*, 2018). Although individual attitudes and beliefs play a role in each of the equity concepts, psychological factors hold a particular importance in recognitional equity given their direct ties to shared understanding and subjectivity (Mao, 2018).

In energy and environmental sociodemographic practices, shared understanding may manifest in researchers becoming aware of environmental injustice findings, energy poverty findings, and how these interplay with technology development and deployment [refer to Tessum *et al.* (2019) and Bednar and Reames (2020) e.g., environmental injustice and energy poverty studies, respectively]. Individual retainment and acceptance of this evidence, however, are found in the tension between objectivity (i.e., the judgment and acceptance of information through appropriate peer or group scrutiny) and subjectivity (i.e., ideas and thoughts that exist outside of objectivity, which are primarily influenced by unscrutinized perceptions) (Ford, 2004; Randall, 2004).

Energy and environmental injustice awareness are only part of the need in achieving recognitional equity. Critically analyzing individual subjectivity (e.g., personal and professional beliefs, attitudes, perceptions, and preconceptions) in the context of this evidence must occur if we are to expect suitable behavior change (Brown, 2010). For example, whites tend to frame racial issues by decentralizing power dynamics in the reproduction of inequity, while characterizing their own successes to personal characteristics without acknowledgment of evident in-group advantages and biases (Ditomaso *et al.*, 2003). This shows how injustice awareness without critical analysis can undermine behavior change.

Furthermore, academic institutions must recognize and tangibly value effective community-based work to facilitate equitable environmental engineering research success and tenure attainment (e.g., evolving the assessment of research impact in academia to include underrepresented community effects and “untraditional” media influences) (Montoya *et al.*, 2020).

**FIG. 1.**
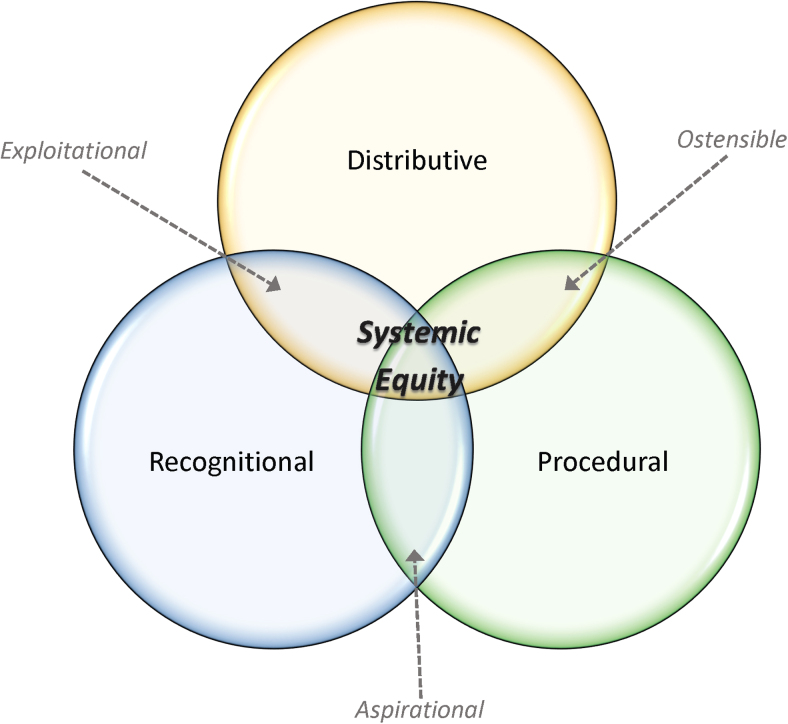
Equity concept applications that must be achieved to realize *systemic equity*, including the terms associated with each subintersectional or two-application union.

Taken together, we posit that *systemic equity* is realized when distributive, procedural, and recognitional equities are simultaneously achieved. While these equity concepts stem from previous literature, the framework for systemic equity and its two-application unions (i.e., the union of distributive and procedural equity, procedural and recognitional equity, and distributive and recognitional equity) are original concepts to the best of our knowledge. Furthermore, this framework aligns with the Peace, Justice and Strong Institutions SDG. Figure 1 illustrates the intersectionality of these concepts.

### Two-application unions for systemic equity

As previously mentioned, there have been many studies that assert equity is integrated into their analyses and decision-making outcomes. However, some of these touted forms of equity can be ineffective in their administration and may cause further harm. To help provide common language and shared understanding in this regard, we provide terminology and concept explanation for the two-application unions of systemic equity (i.e., ostensible, aspirational, and exploitational equity).

*Ostensible equity* occurs when resource (i.e., distributional equity) and policy (i.e., procedural equity) needs are met, but cultural needs (i.e., recognitional equity) are inadequately met. Organizations or systems that exhibit this style of equity may formally establish satisfactory policies and procedures, at least in writing, and provide resources for equitable data collection and processing. However, they tend not to follow through on the activities associated with actual behavior change.

When recognitional aspects are deemed lackluster, systemic equity cannot be achieved. For example, in a community-engaged study that examined coastal environmental justice in Papua New Guinea, feelings of disrespect (e.g., nonadherence to customary norms) were found to undermine the perceived legitimacy of procedural activities and fair distribution (Lau *et al.*, 2021). This same study found that administering satisfactory recognition contributes to the legitimacy of procedural and distributive aspects.

The ineffective administration of recognitional equity can result in ostensible equity whether intentional or not. When there are stakeholders from groups that have historically had more power than the communities they are engaging (e.g., a research laboratory, government entity, or some practitioner group), it is important to rely on the sentiment of those from affected communities and scientific tools to assess the successful application of recognitional aspects. It may even require the development and incorporation of qualitative social science methods (Preston and Carr, 2018). An organization can only achieve recognitional equity when the collective sentiment of the affected community suggests that previous injustices have been appropriately addressed.

*Aspirational equity* occurs when policy (i.e., procedural equity) and cultural needs (i.e., recognitional equity) are met, but resources (i.e., distributive equity) are inadequate. This can be exhibited in organizations that show evidence of developing effective and appropriate procedures, while fostering an authentic and inclusive environment, but they underperform in distributing resulting benefits to vulnerable communities.

It can be argued that organizations and groups that fall into this category possess the want and need to reach systemic equity but lack access to adequate resources. A clear example of this is shown in the tension between environmental justice groups and extractive industries (e.g., the coal power plant and oil industries). The efforts of environmental justice groups have been made more difficult by the historical inequitable distribution of economic resources to extractive industries, in the form of tax breaks or legislative influence, with limited distribution of resources to groups that aim to preserve natural resources and ecosystem services (Malin *et al.*, 2019). A lack of distributive resources and foresight can result in net-negative outcomes and undermine trust between vulnerable communities and their partners (Boone *et al.*, 2009; Schwarz *et al.*, 2015). Nonetheless, the implications of aspirational equity are varied.

*Exploitational equity* occurs when resource (i.e., distributive equity) and cultural needs (i.e., recognitional equity) are met, but policies (i.e., procedural equity) are inadequate. Groups that exhibit this may exude the ideals and principles of equity in their projected messaging while, at least periodically, providing resources to support such ideals. Despite this messaging, they lack the kind of established policies and procedures that systematically yield equitable outcomes. Even well-funded environmental justice initiatives can fail to be tangibly responsive to affected communities when procedural integrity and follow-through wane over time (Heaney *et al.*, 2011; Brown *et al.*, 2018).

Exploitational equity may also emerge when groups that have access to proprietary sociodemographic data (e.g., data acquired through nondisclosure agreement) use such data for professional gain, while failing to make these same data accessible to other groups that may lack similar privilege and access. This could make science that uses such data difficult to replicate or to use in answering new and interesting research questions, thereby undermining the tenets of systemic equity.

The detailed conditions and criteria for the achievement of each equity concept in energy and environmental LCA and decision-making require further exploration, preferably by a broader group of scientists and decision-making stakeholders. This is of particular importance for the two-application unions (i.e., ostensible, aspirational, and exploitational equity), given the risk of not delivering on equity promises.

Furthermore, criteria for systemic equity may change dependent on application context. Relevant criteria that may develop for the energy and environmental community may differ from those of other scientific subfields. Nonetheless, we present an approach to establish conditions and criteria for this joint subfield, in the Standardization of Data Practices section, with the expectation that our concept structure and subsequent guidance will be applied to other subfields in due time.

### Example sociodemographic data needs

Ensuring data inputs are socially relevant is fundamental given that virtually all LCA and decision-making components are influenced by individual human behavior and organizational acts (Fellows, 2004; Gutowski, 2018). We, therefore, argue that systemic equity is only attainable in energy and environmental LCA and decision-making when sociodemographic data are deliberately developed, utilized, or qualified as a normative practice in applicable studies. Achieving this as a normative practice is undermined when there are sociodemographic data gaps that persist.

There are previous works that substantiate the need for sociodemographic data and standards (Jenkins *et al.*, 2021). Nonetheless, we highlight some recent studies to reinforce this substantiation and to provide example sociodemographic needs. Recent works indicate needs in areas such as spatiality and geography (Carley and Konisky, 2020; Fraser and Chapman, 2020; Chapman *et al.*, 2021; Subramanian *et al.*, 2021), urgency for addressing energy and environmental equity and justice matters (Tessum *et al.*, 2019; Bozeman *et al.*, 2020; Wang *et al.*, 2021), industry data that may currently be considered proprietary (Fortier *et al.*, 2019; Tong *et al.*, 2021), improved quality and timescale data (Bozeman *et al.*, 2019, 2020; Chapman *et al.*, 2021), and quantitative methods establishment (Jenkins *et al.*, 2021).

Given these example needs, we were compelled to explore sociodemographic data standardization as a path toward building consensus on quantitative methods in the energy and environmental subfields. As explained in the systematic and comprehensive review performed in Jenkins *et al.* (2021), applicable “justice” research, from years 2008 to 2019, showed a lack of coherent and consistent quantitative collection methodology. Many studies, therein, did not include an explicit methods section. This hinders scientific reproducibility and method refinement. It is also important that sociodemographic data be provided in quantitative terms to allow for easier incorporation into existing energy and environmental methodologies. We talk more about how this can systematically be done next, in the Standardization of Data Practices section.

## Standardization of Data Practices

### Past successes and benefits

There are many examples of data collection standardization successes and benefits for associated practitioner and research activities. Before we describe an apt research example, we begin with two practitioner examples that were facilitated by the International Organization for Standardization (ISO). Both the ISO14001 and 50001 standards are an internationally agreed set of requirements that encompass requirements, including data and record management protocols, the documentation of noncompliance incidents, and the systematic addressing of noncompliance incidents. Although these ISO standards share many components, they differ in their intended practitioner aim. That is, ISO14001 was primarily designed for environmental practitioners, whereas ISO50001 was designed for energy practitioners.

Both ISO standards have facilitated success for their respective practitioner groups. In the case of environmental programming, ISO14001 adherence has been shown to systematically improve organizational cost efficiency, employee productivity, and the return on assets (Treacy *et al.*, 2019). For energy programming, adherence to the ISO50001 requirement of systematically addressing noncompliance incidents has led to tangible data collection and system improvements (Walsh *et al.*, 2015). One study exemplifies this by showing that ISO50001 adherence led to energy savings in manufacturing, while highlighting other organizational issues (e.g., change management programming inefficiencies, lack of leadership buy-in, and ineffective communication strategies) that would otherwise inhibit energy savings (Mohamad *et al.*, 2014).

Research activities have been shown to benefit from standardization practices. The Preferred Reporting Items for Systematic Reviews and Meta-Analyses (PRISMA) is an ideal example of successfully developing and using data collection and presentation standards in research. PRISMA, a framework for reporting on systematic reviews and meta-analyses, was first disseminated in the year 2009 by a group of methodologists, systematic reviewers, clinicians, and journal editors. It included 27 checklist items that established minimum information standards.

PRISMA was updated in the year 2020 after surveying 220 systematic review methodologists and journal editors, facilitating a 2-day in-person meeting of 21 members, and after further refinement given the feedback from coauthors (Page *et al.*, 2021). As a result, the checklist was lengthened, and other standards were consolidated and refined. PRISMA helps to ensure complete and transparent reporting while also facilitating the discovery of reporting biases and the development of complementary standards (Zorzela *et al.*, 2016). An equity-focused extension to PRISMA was even developed (i.e., PRISMA-E), addressing issues around health inequity (Welch *et al.*, 2016).

Taking the findings of these practitioner and research examples together, establishing standards and conditions for sociodemographic data practices in energy and environmental systems is a logical next step. Not doing so would severely limit the attainment of systemic equity in research practices involving LCA and decision-making. The energy and environmental research communities have published enough on matters of justice and equity to suggest a willingness for achieving systemic equity in our spheres of influence. Next, we present a path forward, which aligns with how PRISMA was developed.

### Ten steps for sociodemographic data standardization

To date, we are unaware of any formal and systematic effort to build consensus within the energy and environmental research communities for the standardization of sociodemographic data practices in LCA and decision-making. It follows that a path forward be outlined to fill this gap, given we have established that doing so is vital to achieving systemic equity. Despite previous calls to address data or outcome inequities in energy and environmental decision-making (Reames, 2016; Bozeman *et al.*, 2019; Bednar and Reames, 2020; Jenkins *et al.*, 2021; Tong *et al.*, 2021; Young *et al.*, 2021), none have gone as far as to propose stakeholder inclusion and deliverable conditions.

Table 1 shows an adaptive 10-step process for the systematic standardization of sociodemographic data practices intended to address development, collection, and reporting norms. It is expected that this will also facilitate systemic equity within energy and environmental LCA and decision-making. To be effective at each step requires committed and persistent stakeholders from various disciplines and geographic regions. Furthermore, it will require the kind of support that satisfies each equity concept (i.e., distributive, procedural, and recognitional equity) and an alignment with the multistakeholder partnership provisions in the Partnerships for the Goals SDG.

**Table 1.** Ten Steps for the Standardization of Sociodemographic Data Practices

Step no.	Action	Deliverable(s)
1	Identify and confirm equity-focused energy and environmental professionals	Initial planning group is formed
2	Identify and confirm collaborative transdisciplinary professionals	Survey planning and preliminary development group is formed
3	Develop preliminary systemic equity checklist and criteria for sociodemographic data practices	Preliminary survey framework and checklist/criteria are formed
4	Develop and administer survey to a predetermined amount of equity-focused professionals, seeking input	Survey responses inform the in-person focus group framework and checklist/criteria
5	Assess and incorporate survey responses during an in-person focus group of equity-focused professionals	Finalized checklist/criteria are formed
6	Submit findings to accessible outlets and make checklist/criteria public	Finalized checklist/criteria are promulgated
7	Identify and confirm equity champions for each global region	Establishes a global communication and checklist/criteria dissemination framework
8	Develop a follow-up survey to assess checklist/criteria effectiveness and satisfaction	Survey responses inform checklist/criteria refinement
9	Assess and incorporate survey responses during an in-person meeting of equity-focused professionals	Updated checklist/criteria are established
10	Repeat Steps 6 through 9 periodically given a concurred period (e.g., every 5 years)	Establishes a systemic equity process

Satisfying these steps will require robust transdisciplinary collaboration. Steps One through Five call for the formation of a group made up of energy, environmental, and transdisciplinary stakeholders. These include professional types such as researchers, practitioners (e.g., government entities and policy makers), and journal editors. We provide inclusion guidance for each of these example stakeholder types in the subsequent paragraphs.

Ideal researchers should have a record or serious interest in LCA or decision-making matters that advance equity. It is important to note that there are a multitude of methodologies used in decision-making. Therefore, the researchers who join this initial effort can have methodological expertise that differs from those who primarily use LCA, so as long as the methodological approach is recognized as impacting decision-making. Industrial ecology methodologies (e.g., material flow analysis, scenario analysis, LCA, urban metabolism, and economic input–output) serve as an example of research approaches that tend to influence decision-making (Thomas *et al.*, 2003; Meerow and Newell, 2015).

Social scientists are an example of an ideal cross-disciplinary collaborator. Their involvement can bolster the development and administration of associated surveys, checklists, and criteria. It has already been established that addressing matters of equity and justice requires the collaboration of various scientific disciplines. We further substantiate cross-disciplinary engagement by emphasizing that many social science subfields administer surveys and questionnaires as a core approach. We suggest that professionals from the public health, social psychology, and economic subfields be involved throughout each step after Step One. Such engagement would bolster efforts to reach diverse audiences in the survey activities described in Steps Four and Eight.

Involving practitioners is vital to the success of this effort. There are several systems where practitioners influence sociodemographic data access and decision-making. For instance, Bozeman, Bozeman, and Theis (2020) performed an LCA investigation using sociodemographic data collected by the US Department of Agriculture (USDA) that yielded national-level food consumption impacts on land, greenhouse gas, and water across racial and ethnic subgroups (i.e., white, black, and Latinx). The USDA administers periodic surveys on food consumption across SES and racial and ethnic subgroups. The data from these surveys were made available through a joint effort between them and the University of Maryland via a database called the Environmental Protection Agency Food Commodity Intake Database.

In another study example, the US Bureau of Labor Statistics provided consumer expenditure survey data across SES and racial and ethnic subgroups (Bozeman *et al.*, 2019). Government groups such as these are staffed by many practitioners. The way these professionals collect and disseminate sociodemographic data has direct impact on researchers' abilities to equitably inform decision-making.

Government entities are not the only ones influencing sociodemographic data practices and decision-making. Private companies, and their associated practitioners, also influence the availability and accessibility of useful data. Furthermore, community groups, although they too often experience inequitable power in decision-making, have influence on sociodemographic data access and decision-making outcomes.

Journal editors are a key stakeholder group. They can influence article submission standards. Once the checklist that derives from following Table 1 is finalized, journal editors, especially from publications that support equity- and justice-centered submissions, could require that the completed checklist accompanies all author submissions unless otherwise justified. This aligns itself to how PRISMA is administered. Some journals that focus on publishing systematic reviews or meta-analyses insist that PRISMA, or another explicated framework with similar components, be completed and accompany initial article submissions (Tam *et al.*, 2017).

Steps Six through Nine are to be repeated on a cyclical basis (e.g., every 5 years) as a condition of Step Ten. Social challenges evolve; thus, it is vital that we ensure these standards are periodically refined to match the disposition of the equity methods and transdisciplinary approaches that emerge over time. Furthermore, there will likely need to be periodic assessment of funding and organizational support to meet the requirements of systemic equity.

Sociodemographic framing may differ from region to region. For example, the categorization of racial and ethnic subgroups in North American countries may not translate directly to that of other global regions. Therefore, Step Seven indicates that equity champions from different sociodemographic subgroups be established to assist in promoting the finalized standards in their respective global regions.

### Special considerations for the 10-step process

We highlight several important factors that should be addressed as part of the 10-step process. This process is an adaptive one, in that the standards that emerge will likely vary in applicability and administration conditions. For instance, the users of these standards are not clearly defined given the present study establishes a novel process for achieving standardization and does not establish the standards themselves.

The actual standards will not take form until Step Five yields its key deliverable (i.e., a finalized checklist/criteria). Therefore, determining which specific groups outside of energy and environmental stakeholders will use these to-be-developed standards, how long it will take each step to occur, how these standards will be tangibly applied to various LCA stages and spatial scales, and how the culmination of these factors will affect decision-making is outside the scope of the present study.

This 10-step process is intended to provide a framework for addressing each of these factors over time, although it may take more than one 10-step iteration to address them comprehensively.

There is also the potential that collecting and promulgating certain kinds of sociodemographic data could be harmful to the systematically marginalized. For example, caution must be used when human subjects such as undocumented workers or those who identify in a manner that may have social implications (e.g., sexual orientation, religious affiliation, and other forms of personal identifiers) are incorporated into data collection or promulgation activities. In the case of survey administration, internal review board proceedings are intended to address potential conflicts in participant data management and harmful collection practices (Grady, 2019). Nonetheless, it is important that those involved in this 10-step process consider human subject sensitivities carefully.

Lastly, there may be other innovations that manifest during the 10-step process that are not explicated in the present study. For example, Step Seven could facilitate the establishment of country-level champions, in addition to the global-regional-level equity champions, if time and resources permit. There are many other potentials that could emerge when following this line of thinking.

## Conclusion and Discussion

In this article, we sought to establish initial steps toward standardizing sociodemographic data practices in energy and environmental LCA and decision-making. In doing so, we presented a framework for achieving systemic equity (i.e., the simultaneous administration of distributive, procedural, and exploitational equity), provided terminology and concept explanation for the two-application unions of systemic equity (i.e., ostensible, aspirational, and exploitational equity), and established an adaptive 10-step process for standardizing sociodemographic data practices. The energy and environmental scientific communities, in concert with transdisciplinary partners, should perform these standardization steps with a sense of urgency.

### S-LCA implications

As established in the Introduction section, S-LCA explicitly incorporates social and subjective components. As such, its evolution has the potential to help organizations meet systemic equity. For example, Fortier *et al.* (2019) introduced S-LCA indicators that address energy justice. These indicators, if combined with other LCA tools that address equity and justice matters, may evolve to form more comprehensive tools that can assess if the tenets of systemic equity are adequately integrated. Such tools are less likely to manifest across disciplines without frequent and relatively easy access to sociodemographic data. This reinforces the urgency around using the present study's 10-step process for standardizing sociodemographic data practices.

## References

[B1] Adeyeye, Y., Hagerman, S., and Pelai, R. (2019). Seeking procedural equity in global environmental governance: Indigenous participation and knowledge politics in forest and landscape restoration debates at the 2016 World Conservation Congress. For. Policy Econ. 109, 102006.

[B2] Bai, X., Dawson, R.J., Ürge-Vorsatz, D., Delgado, G.C., Salisu Barau, A., Dhakal, S., Dodman, D., Leonardsen, L., Masson-Delmotte, V., Roberts, D.C., and Schultz, S. (2018). Six research priorities for cities and climate change. Nature (London). 555, 23. 10.1038/d41586-018-02409-z 29493611

[B3] Baker, E., Goldstein, A.P., and Azevedo, I.M.L. (2021). A perspective on equity implications of net zero energy systems. Energy Clim. Change. 2, 100047.

[B4] Baker, E., Nock, D., Levin, T., Atarah, S.A., Afful-Dadzie, A., Dodoo-Arhin, D., Ndikumana, L., Shittu, E., and Muchapondwai, E., Sackey, C.V.-H. (2021). Who is marginalized in energy justice? Amplifying community leader perspectives of energy transitions in Ghana. Energy Res. Soc. Sci. 73, 101933.

[B5] Baker, S., DeVar, S., and Prakash, S. (2019). The energy justice workbook. In: Initiative for Energy Justice. Available at: https://www.cebrightfutures.org/sites/default/files/resource-files/The-Energy-Justice-Workbook-2019-web.pdf (Accessed March 30, 2022).

[B6] Balsiger, J. (2011). The art and craft of international environmental law—By Daniel Bodansky. Rev. Policy Res. 28, 382.

[B7] Baltruszewicz, M., Steinberger, J.K., Owen, A., Brand-Correa, L.I., and Paavola, J. (2021). Final energy footprints in Zambia: Investigating links between household consumption, collective provision, and well-being. Energy Res. Soc. Sci. 73, 101960.

[B8] Barber, P.H., Hayes, T.B., Johnson, T.L., Márquez-Magaña, L., and signatories. (2020). Systemic racism in higher education. Science. 369, 1440. 10.1126/science.abd7140 32943517

[B9] Bednar, D.J., and Reames, T.G. (2020). Recognition of and response to energy poverty in the United States. Nat. Energy. 5, 432. 10.1016/j.erss.2023.103045 PMC10049889 37006444

[B10] Benoit Norris, C. (2013). Data for social LCA. Int. J. Life Cycle Assess. 19, 261.

[B11] Biden, J.R. (2021). Tackling the Climate Crisis at Home and Abroad. Federal Register: Presidential Documents. Available at: https://www.whitehouse.gov/briefing-room/presidential-actions/2021/01/27/executive-order-on-tackling-the-climate-crisis-at-home-and-abroad/ (Accessed March 30, 2022).

[B12] Boone, C.G., Buckley, G.L., Grove, J.M., and Sister, C. (2009). Parks and people: An environmental justice inquiry in Baltimore, Maryland. Ann. Assoc. Am. Geogr. 99, 767.

[B13] Bozeman, J.F., Ashton, W.S., and Theis, T.L. (2019). Distinguishing environmental impacts of household food-spending patterns among U.S. demographic groups. *Environ. Eng. Sci*. 36, 763.

[B14] Bozeman, J.F., Bozeman, R., and Theis, T.L. (2020). Overcoming climate change adaptation barriers: A study on food–energy–water impacts of the average American diet by demographic group. J. Ind. Ecol. 24, 383.

[B15] Bozeman, J.F., Springfield, S., and Theis, T.L. (2020). Meeting EAT-lancet food consumption, nutritional, and environmental health standards: A U.S. case study across racial and ethnic subgroups. *Environ. Just*. 13, 160. 10.1089/env.2020.0018 PMC7580058 33101580

[B16] Brown, K. (2010). Schools of Excellence and Equity? Using Equity Audits as a Tool to Expose a Flawed System of Recognition. Int. J. Educ. Policy Leadersh. 5, 1.

[B17] Brown, P., Vega, C.M.V., Murphy, C.B., Welton, M., Torres, H., Rosario, Z., , Alshawabkeh, A., Cordero, J.F., Padilla, I.Y. and Meeker, J. D. (2018). Hurricanes and the environmental justice Island: Irma and Maria in Puerto Rico. Environ. Just. 11, 148. 10.1089/env.2018.0003 PMC6114726 31131071

[B18] Carley, S., and Konisky, D.M. (2020). The justice and equity implications of the clean energy transition. Nat. Energy. 5, 569.

[B19] Chapman, A., Shigetomi, Y., and Ohno, H. (2021). Renewable Energy Investments and Social Equity: Evaluating the Low-Carbon Energy Transition. Available at: https://papers.ssrn.com/sol3/papers.cfm?abstract_id=3807630 (Accessed March 30, 2022).

[B20] Conrad, Z., Niles, M.T., Neher, D.A., Roy, E.D., Tichenor, N.E., and Jahns, L. (2018). Relationship between food waste, diet quality, and environmental sustainability. PLoS One. 13, e0195405. 29668732 10.1371/journal.pone.0195405 PMC5905889

[B21] Diotalevi, R.N., and Burhoe, S. (2017). Native American lands and the Keystone Pipeline Expansion: A legal analysis. Ind. Policy J. 27.

[B22] Ditomaso, N., Parks-Yancy, R., and Post, C. (2003). White views of civil rights: Color blindness and equal opportunity. In: Routledge, pp. 190–199. Available at: https://www.taylorfrancis.com/chapters/edit/10.4324/9780203412107-22/white-views-civil-rights-color-blindness-equal-opportunity-nancy-ditomaso-rochelle-parks-yancy-corinne-post (Accessed March 30, 2022).

[B23] Doyle, M.W., and Stiglitz, J.E. (2014). Eliminating extreme inequality: A sustainable development goal, 2015–2030. Ethics Int. Aff. 28, 5.

[B24] Earles, J.M., and Halog, A. (2011). Consequential life cycle assessment: A review. Int. J. Life Cycle Assess. 16, 445.

[B25] Ekvall, T. (2000). Moral philosophy, economics, and life cycle inventory analysis. SAE Tech. Papers. DOI: 10.4271/2000-01-1479.

[B26] EPA. (2021). Environmental Justice. Washington, DC: United States Environmental Protection Agency: United States Environmental Protection Agency.

[B27] Fellows, L.K. (2004). The cognitive neuroscience of human decision making: A review and conceptual framework. Behav. Cogn. Neurosci. Rev. 3, 159. 15653813 10.1177/1534582304273251

[B28] Finnveden, G. (2000). On the limitations of life cycle assessment and environmental systems analysis tools in general. Int. J. Life Cycle Assess. 5, 229.

[B29] Ford, N. (2004). Creativity and convergence in information science research: The roles of objectivity and subjectivity, constraint, and control. J. Am. Soc. Inform. Sci. Technol. 55, 1169.

[B30] Fortier, M.-O.P., Teron, L., Reames, T.G., Munardy, D.T., and Sullivan, B.M. (2019). Introduction to evaluating energy justice across the life cycle: A social life cycle assessment approach. Appl. Energy 236, 211.

[B31] Fraser, T., and Chapman, A.J. (2020). Drivers of social equity in renewable energy at the municipal level: The case of local Japanese energy policy and preferences. J. Environ. Policy Plan. 22, 397.

[B32] Freistein, K., and Mahlert, B. (2016). The potential for tackling inequality in the Sustainable Development Goals. Third World Q. 37, 2139.

[B33] Friedman, R.S., Law, E.A., Bennett, N.J., Ives, C.D., Thorn, J.P.R., and Wilson, K.A. (2018). How just and just how? A systematic review of social equity in conservation research. Environ. Res. Lett. 13, 053001.

[B34] Grady, C. (2019). The contribution of ethics review to protection of human participants: Comment on “measuring the quality and performance of institutional review boards.” J. Empir. Res. Hum. Res. Ethics. 14, 197. 30896324 10.1177/1556264619837774

[B35] Gurram, S., Stuart, A.L., and Pinjari, A.R. (2019). Agent-based modeling to estimate exposures to urban air pollution from transportation: Exposure disparities and impacts of high-resolution data. Comput. Environ. Urban Syst. 75, 22.

[B36] Gutowski, T.G. (2018). A critique of life cycle assessment; where are the people? *Proc*. CIRP. 69, 11.

[B37] Guy, M.E., and McCandless, S.A. (2012). Social equity: Its legacy, its promise. Public Adm. Rev. 72(s1), S5.

[B38] Hamann, M., Berry, K., Chaigneau, T., Curry, T., Heilmayr, R., Henriksson, P.J.G., Hentati-Sundberg, J., Jina, A., Lindkvist, E., Lopez-Maldonado, Y., Nieminen, E., Piaggio, M., Qiu, J., Rocha, J.C., Schill, C., Shepon, A., Tilman, A.R., van den Bijgaart, I., and Wu, T. (2018). Inequality and the biosphere. Ann. Rev. Environ. Resour. 43, 61.

[B39] Hartman, S.J., Yrle, A.C., and Galle, W.P. (1999). Procedural and distributive justice: Examining equity in a university setting. J. Bus. Ethics. 20, 337.

[B40] Havard, S., Deguen, S., xe, verine, Zmirou-Navier, D., Schillinger, C., and Bard, D. (2009). Traffic-related air pollution and socioeconomic status: A spatial autocorrelation study to assess environmental equity on a small-area scale. Epidemiology. 20, 223. 19142163 10.1097/EDE.0b013e31819464e1

[B41] Heaney, C., Wilson, S., Wilson, O., Cooper, J., Bumpass, N., and Snipes, M. (2011). Use of community-owned and -managed research to assess the vulnerability of water and sewer services in marginalized and underserved environmental justice communities. J. Environ. Health. 74, 8. 21830685

[B42] Hearst, M.O., Sirard, J.R., Forsyth, A., Parker, E.D., Klein, E.G., Green, C.G., and Lytle, L.A. (2013). The relationship of area-level sociodemographic characteristics, household composition and individual-level socioeconomic status on walking behavior among adults. Transp. Res. Part A Policy Pract. 50, 149. 23729994 10.1016/j.tra.2013.01.006 PMC3667602

[B43] Heffron, R.J., and McCauley, D. (2018). What is the ‘just transition’? Geoforum. 88, 74.

[B44] Hellweg, S., and Milà i Canals, L. (2014). Emerging approaches, challenges and opportunities in life cycle assessment. Science. 344, 1109. 24904154 10.1126/science.1248361

[B45] Ikeme, J. (2003). Equity, environmental justice and sustainability: Incomplete approaches in climate change politics. Glob. Environ. Change. 13, 195.

[B46] Jenkins, K., McCauley, D., Heffron, R., Stephan, H., and Rehner, R. (2016). Energy justice: A conceptual review. Energy Res. Soc. Sci. 11, 174.

[B47] Jenkins, K., Sovacool, B.K., and McCauley, D. (2018). Humanizing sociotechnical transitions through energy justice: An ethical framework for global transformative change. Energy Policy. 117, 66.

[B48] Jenkins, K.E.H., Sovacool, B.K., Mouter, N., Hacking, N., Burns, M.-K., and McCauley, D. (2021). The methodologies, geographies, and technologies of energy justice: A systematic and comprehensive review. Environ. Res. Lett. 16, 043009.

[B49] Jenkins, K.E.H., Stephens, J.C., Reames, T.G., and Hernández, D. (2020). Towards impactful energy justice research: Transforming the power of academic engagement. Energy Res. Soc. Sci. 67, 101510.

[B50] Jordaan, S.M., Combs, C., and Guenther, E. (2021). Life cycle assessment of electricity generation: A systematic review of spatiotemporal methods. Adv. Appl. Energy. 3, 100058.

[B51] Kaab, A., Sharifi, M., Mobli, H., Nabavi-Pelesaraei, A., and Chau, K.-W. (2019). Combined life cycle assessment and artificial intelligence for prediction of output energy and environmental impacts of sugarcane production. Sci. Total Environ. 664, 1005. 30769303 10.1016/j.scitotenv.2019.02.004

[B52] Klein, S.J.W., and Whalley, S. (2015). Comparing the sustainability of U.S. electricity options through multi-criteria decision analysis. *Energy Policy.* 79, 127.

[B53] Klöpffer, W. (2012). The critical review of life cycle assessment studies according to ISO 14040 and 14044. Int. J. Life Cycle Assess. 17, 1087.

[B54] Lau, J.D., Gurney, G.G., and Cinner, J. (2021). Environmental justice in coastal systems: Perspectives from communities confronting change. Glob. Environ. Change. 66, 102208.

[B55] Lazarevic, D., and Martin, M. (2016). Life cycle assessments, carbon footprints and carbon visions: Analysing environmental systems analyses of transportation biofuels in Sweden. J. Clean. Prod. 137, 249.

[B56] Lisovich, M.A., and Wicker, S.B. (2008). Privacy concerns in upcoming residential and commercial demand-response systems. IEEE Proc. Power Syst. 1, 1.

[B57] Malin, S.A., Ryder, S., and Lyra, M.G. (2019). Environmental justice and natural resource extraction: Intersections of power, equity and access. Environ. Sociol. 5, 109.

[B58] Mao, Y. (2018). The impact of affect on organizational justice perceptions: A test of the affect infusion model. J. Manag. Organ. 24, 893.

[B59] McLean, M. (2016). How smart is too smart? How privacy concerns threaten modern energy infrastructure. Vand. J. Ent. Technol. Law. 18, 879.

[B60] Meerow, S., and Newell, J.P. (2015). Resilience and complexity: A bibliometric review and prospects for industrial ecology. J. Ind. Ecol. 19, 236.

[B61] Mistretta, M., Caputo, P., Cellura, M., and Cusenza, M.A. (2019). Energy and environmental life cycle assessment of an institutional catering service: An Italian case study. Sci. Total Environ. 657, 1150. 30677882 10.1016/j.scitotenv.2018.12.131

[B62] Mohamad, F., Abdullah, N.H., Kamaruddin, N.K., and Mohammad, M. (2014). Implementation of ISO50001 energy management system. In: 2014 International Symposium on Technology Management and Emerging Technologies.

[B63] Montoya, L.D., Mendoza, L.M., Prouty, C., Trotz, M., and Verbyla, M.E. (2020). Environmental engineering for the 21st century: Increasing diversity and community participation to achieve environmental and social justice. Environ. Eng. Sci. 38, 288. 10.1089/ees.2020.0148 PMC8165463 34079202

[B64] Niessen, L.W., Mohan, D., Akuoku, J.K., Mirelman, A.J., Ahmed, S., Koehlmoos, T. P.,. Trujillo, A., Khan, J., Peters, D.H. (2018). Tackling socioeconomic inequalities and non-communicable diseases in low-income and middle-income countries under the Sustainable Development agenda. Lancet (Br. Ed.). 391, 2036. 10.1016/S0140-6736(18)30482-3 29627160

[B65] Nock, D., and Baker, E. (2019). Holistic multi-criteria decision analysis evaluation of sustainable electric generation portfolios: New England case study. Appl. Energy. 242, 655.

[B66] Page, M.J., McKenzie, J.E., Bossuyt, P.M., Boutron, I., Hoffmann, T.C., Mulrow, C. D., Shamseer, L., Tetzlaff, J.M., Moher, D. (2021). Updating guidance for reporting systematic reviews: Development of the PRISMA 2020 statement. J. Clin. Epidemiol. 134, 103. 33577987 10.1016/j.jclinepi.2021.02.003

[B67] Pfeiffer, O., Nock, D., and Baker, E. (2021). Wind energy's bycatch: Offshore wind deployment impacts on hydropower operation and migratory fish. Renew. Sustain. Energy Rev. 143, 110885.

[B68] Preston, C., and Carr, W. (2018). Recognitional justice, climate engineering, and the care approach. Ethics Policy Environ. 21, 308.

[B69] Pulido, L. (1996). A critical review of the methodology of environmental racism research. Antipode. 28, 142.

[B70] Randall, M. (2004). “Unsubstantiated belief”: What we assume as truth, and how we use those assumptions. J. Am. Folklore. 117, 288.

[B71] Reames, T.G. (2016). Targeting energy justice: Exploring spatial, racial/ethnic and socioeconomic disparities in urban residential heating energy efficiency. Energy Policy. 97, 549.

[B72] Romero-Lankao, P., Fritz, S., and Supple, L. (2021). The Social, Institutional, and Cultural Dimensions of Energy Transitions: A National Renewable Energy Lab (NREL) Accelerating Clean Energy at Scale (ACES) External Workshop & Partnership Development Series. Available at: https://www.nrel.gov/state-local-tribal/aces.html

[B73] Romero-Lankao, P., and Nobler, E. (2021). Energy Justice: Key Concepts and Metrics Relevant to EERE Transportation Projects. *National Renewable Energy Laboratory.* Management Report, NREL/TP-5400-80206. Available at: https://www.osti.gov/biblio/1797919

[B74] Schwarz, K., Fragkias, M., Boone, C.G., Zhou, W., McHale, M., Grove, J.M., O'Neil-Dunne, J., McFadden, J.P., Buckley, G.L., Childers, D., Ogden, L., Pincetl, S., Pataki, D., Whitmer, A., Cadenasso, M.L., Loiselle, S.A. (2015). Trees grow on money: Urban tree canopy cover and environmental justice. PLoS One. 10, e0122051. 25830303 10.1371/journal.pone.0122051 PMC4382324

[B75] Sobal, J. (1991). Obesity and socioeconomic status: A framework for examining relationships between physical and social variables. Med. Anthropol. 13, 231. 1961104 10.1080/01459740.1991.9966050

[B76] Solomon, B.D., and Krishna, K. (2011). The coming sustainable energy transition: History, strategies, and outlook. Energy Policy. 39, 7422.

[B77] Sovacool, B.K., Heffron, R.J., McCauley, D., and Goldthau, A. (2016). Energy decisions reframed as justice and ethical concerns. Nat. Energy. 1, 16024.

[B78] Sovacool, B.K., Hook, A., Martiskainen, M., and Baker, L. (2019). The whole systems energy injustice of four European low-carbon transitions. Glob. Environ. Change. 58, 101958.

[B79] Stevens, K.R., Masters, K.S., Imoukhuede, P.I., Haynes, K.A., Setton, L.A., Cosgriff-Hernandez, E., Lediju Bell, M.A., Rangamani, P., Sakiyama-Elbert, S.E., Finley, S.D., Willits, R.K., Koppes, A.N., Chesler, N.C., Christman, K.L., Allen, J.B., Wong, J.Y., El-Samad, H., Desai, T.A., Eniola-Adefeso, O. (2021). Fund Black scientists. Cell. 184, 561. 33503447 10.1016/j.cell.2021.01.011

[B80] Subramanian, K., Chopra, S.S., Cakin, E., Liu, J., and Xu, Z. (2021). Advancing neighbourhood sustainability assessment by accounting for sustainable development goals: A case study of Sha Tin neighbourhood in Hong Kong. Sustain. Cities Soc. 66, 102649.

[B81] Tam, W.W.S., Lo, K.K.H., and Khalechelvam, P. (2017). Endorsement of PRISMA statement and quality of systematic reviews and meta-analyses published in nursing journals: A cross-sectional study. BMJ Open. 7, e013905. 10.1136/bmjopen-2016-013905 PMC5306529 28174224

[B82] Terpstra, D.E., and Honoree, A.L. (2003). The relative importance of external, internal, individual and procedural equity to pay satisfaction: Procedural equity may be more important to employees than organizations believe. Compens. Benefits Rev. 35, 67.

[B83] Tessum, C.W., Apte, J.S., Goodkind, A.L., Muller, N.Z., Mullins, K.A., Paolella, D. A.,. .. Hill, J. D. (2019). Inequity in consumption of goods and services adds to racial–ethnic disparities in air pollution exposure. Proc. Natl. Acad. Sci. 116, 6001. 30858319 10.1073/pnas.1818859116 PMC6442600

[B84] Thomas, V., Theis, T., Lifset, R., Grasso, D., Byung, K.I.M., Koshland, C., and Pfahl, R. (2003). Industrial ecology: Policy potential and research needs. Environ. Eng Sci. 20, 1.

[B85] Tong, K., Ramaswami, A., Xu, C., Feiock, R., Schmitz, P., and Ohlsen, M. (2021). Measuring social equity in urban energy use and interventions using fine-scale data. Proc. Natl. Acad. Sci. 118, e2023554118. 34099569 10.1073/pnas.2023554118 PMC8214702

[B86] Treacy, R., Humphreys, P., McIvor, R., and Lo, C. (2019). ISO14001 certification and operating performance: A practice-based view. Int. J. Prod. Econ. 208, 319.

[B87] Walsh, B.P., Murray, S.N., and O'Sullivan, D.T.J. (2015). The water energy nexus, an ISO50001 water case study and the need for a water value system. Water Resour. Ind. 10, 15.

[B88] Wang, J.-J., Jing, Y.-Y., Zhang, C.-F., and Zhao, J.-H. (2009). Review on multi-criteria decision analysis aid in sustainable energy decision-making. Renew. Sustain. Energy Rev. 13, 2263.

[B89] Wang, Q., Kwan, M.-P., Fan, J., and Lin, J. (2021). Racial disparities in energy poverty in the United States. Renew. Sustain. Energy Rev. 137, 110620.

[B90] Weidema, B.P., Pizzol, M., Schmidt, J., and Thoma, G. (2018). Attributional or consequential Life Cycle Assessment: A matter of social responsibility. J. Clean. Produc. 174, 305.

[B91] Welch, V., Petticrew, M., Petkovic, J., Moher, D., Waters, E., White, H.,. .. Wells, G. (2016). Extending the PRISMA statement to equity-focused systematic reviews (PRISMA-E 2012): Explanation and elaboration. J. Clin. Epidemiol. 70, 68. 26348799 10.1016/j.jclinepi.2015.09.001

[B92] Williams, D.R. (1996). Race/Ethnicity and socioeconomic status: Measurement and methodological issues. Int. J. Health Serv. 26, 483. 8840198 10.2190/U9QT-7B7Y-HQ15-JT14

[B93] Winnifred, R.B.G. (2010). But some of us are brave: Black women faculty transforming the academy. Signs J. Women Cult. Soc. 35, 801.

[B94] Wood, B.S., and Horner, M.W. (2016). Understanding accessibility to snap-accepting food store locations: Disentangling the roles of transportation and socioeconomic status. Appl. Spat. Anal. Policy. 9, 309.

[B95] Young, S.D., Mallory, B., and McCarthy, G. (2021). Interim Implementation Guidance for the Justice40 Initiative. Executive Office of the President: Executive Office of the President. Available at: https://www.whitehouse.gov/wp-content/uploads/2021/07/M-21-28.pdf (Accessed March 30, 2022).

[B96] Zamagni, A., Amerighi, O., and Buttol, P. (2011). Strengths or bias in social LCA? *Int*. J. Life Cycle Assess. 16, 596.

[B97] Zorzela, L., Loke, Y.K., Ioannidis, J.P., Golder, S., Santaguida, P., Altman, D.G., , Moher, D., Vohra, S. (2016). PRISMA harms checklist: Improving harms reporting in systematic reviews. BMJ. 352, i157. 26830668 10.1136/bmj.i157

